# Metagenome of a Bronchoalveolar Lavage Fluid Sample from a Confirmed COVID-19 Case in Quito, Ecuador, Obtained Using Oxford Nanopore MinION Technology

**DOI:** 10.1128/MRA.00996-20

**Published:** 2020-10-08

**Authors:** Sully Márquez, Belén Prado-Vivar, Juan José Guadalupe, Bernardo Gutierrez, Mónica Becerra-Wong, Manuel Jibaja, Milton Tobar, Verónica Barragán, Patricio Rojas-Silva, Josefina Coloma, Gabriel Trueba, Michelle Grunauer, Paúl Cárdenas

**Affiliations:** a Universidad San Francisco de Quito, COCIBA, Instituto de Microbiología, Quito, Ecuador; b Universidad San Francisco de Quito, Centro de Bioinformática, Quito, Ecuador; c Universidad San Francisco de Quito, Escuela de Medicina, COCSA, Quito, Ecuador; d Universidad San Francisco de Quito, COCIBA, Laboratorio de Biotecnología Vegetal, Quito, Ecuador; e Unidad de Cuidados Intensivos, Hospital Eugenio Espejo, Quito, Ecuador; f Department of Zoology, University of Oxford, Oxford, United Kingdom; g University of California at Berkeley, Berkeley, California, USA; h Unidad de Cuidados Intensivos, Hospital de los Valles, Quito, Ecuador; DOE Joint Genome Institute

## Abstract

We report the metagenome analysis of a bronchoalveolar lavage (BAL) fluid sample from a confirmed coronavirus disease 2019 (COVID-19) case in Quito, Ecuador. Sequencing was performed using MinION technology.

## Abstract (precis)

We report the metagenome analysis of a bronchoalveolar lavage (BAL) fluid sample from a confirmed COVID-19 case in Quito, Ecuador. Sequencing was performed using MinION technology.

## ANNOUNCEMENT

Metagenome analysis could be relevant in critically ill coronavirus disease 2019 (COVID-19) patients. These data can help identify coinfections and provide information for optimal treatment. We collected a sample of bronchoalveolar lavage (BAL) fluid from a confirmed COVID-19 case in Quito, Ecuador (HEE1). The patient, a tourist of Dutch origin in his late 50s, presented respiratory symptoms, including fever and cough, during a visit to Sucumbios Province in Ecuador’s Amazon region. He was admitted with paroxysmal coughing to a public hospital in Lago Agrio, Ecuador, and an initial diagnosis of bacterial pneumonia was made. Diagnosis of COVID-19 was confirmed by the Ecuadorian Ministry of Health on 7 March 2020. Because the patient’s condition deteriorated, he was transferred to the Eugenio Espejo Hospital (HEE) intensive care unit (ICU) in Quito, Ecuador. On 11 March, a nonbronchoscopic protected BAL was performed using the double-catheter technique (the amount of aspirated fluid was 7 ml), and the sample was immediately transported for analysis. Sample positivity to severe acute respiratory syndrome coronavirus 2 (SARS-CoV-2) was confirmed with reverse transcription-quantitative PCR (RT-PCR) using the Veri-Q PCR 316 kit (MiCo BioMed, South Korea) that targets the *ORF3a* and *N* genes; the test came back positive for gene *ORF3a* with a quantification cycle (*C_q_*) value of 32.59. The patient recovered after 1 month of hospitalization and returned to his native country on 10 April.

Metagenome sequencing of the BAL sample was carried out using Oxford Nanopore MinION technology. Total RNA was extracted from 250 μl of the BAL sample using a QIAamp viral RNA extraction kit (Qiagen, Germany) following the manufacturer’s instructions; no DNase digestion step was added. The sample was eluted in a final volume of 70 μl. Extracted RNA was purified using the RNA Clean and Concentrator kit (Zymo Research, USA). Purified RNA (14 μl) was used for retrotranscription of RNA to cDNA following the RNA Viral Metagenomics MinION one-pot sequencing protocol from the genomics department of Public Health England (1, 2). cDNA library preparation was performed using the rapid barcoding kit (SQK-RBK004; Oxford Nanopore Technologies [ONT], UK) following the manufacturer’s instructions. The resulting library was loaded onto an Oxford MinION flow cell (FLO-MIN 106) and sequenced using MinKNOW version 4.05 for 24 h. Base calling and quality control analyses were performed using Guppy version 3.4.5 in high-accuracy mode and NanoPlot version 1.29.0, respectively (3). Adapters and barcodes were removed from the reads using Porechop version 0.2.4 (https://github.com/rrwick/Porechop).

**FIG 1:**
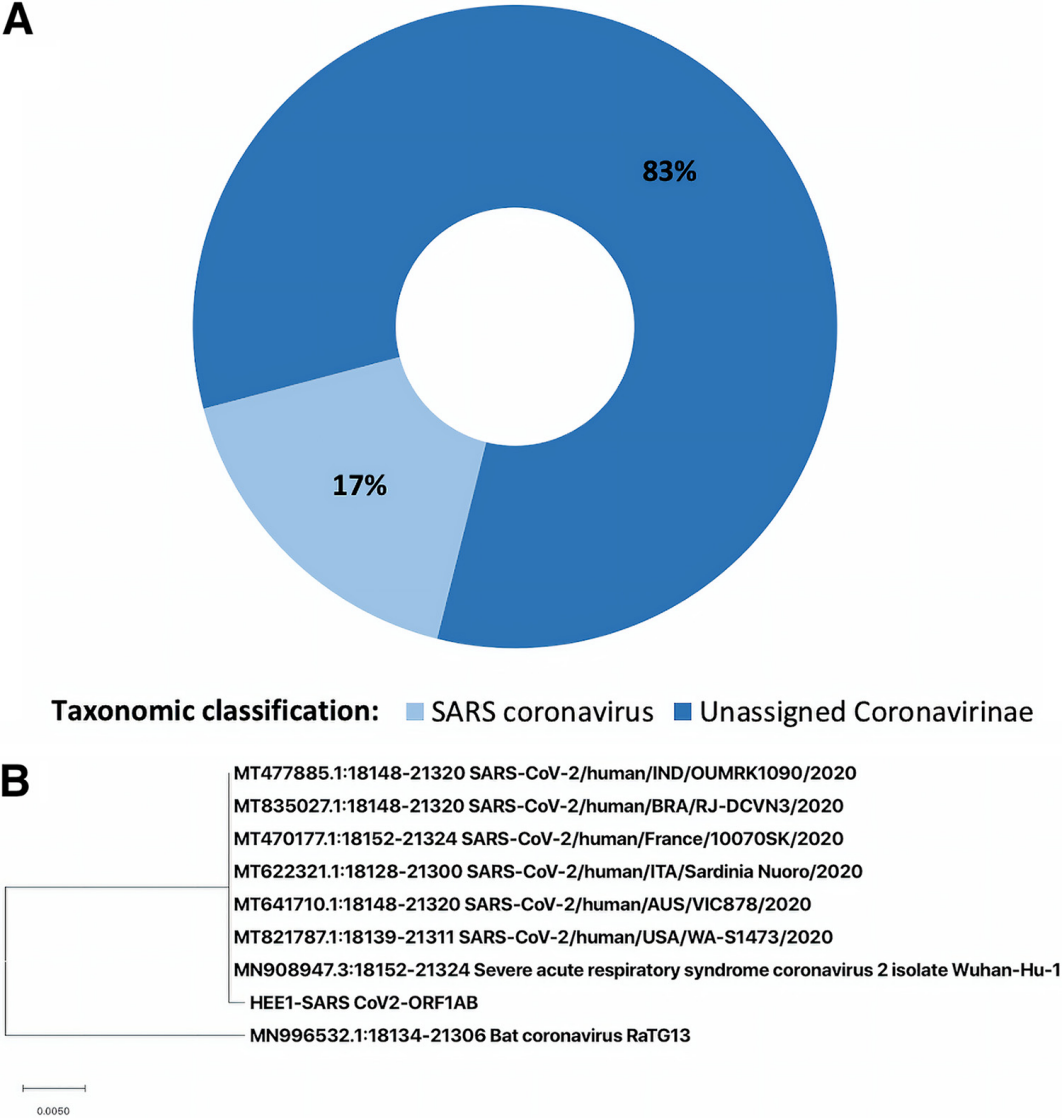
Taxonomic classification of viral sequences recovered from the bronchoalveolar lavage (BAL) fluid metagenome. (A) Krona chart showing the classification of sequences identified as *Coronavirinae*; 83% were unidentified coronaviruses, and 17% were SARS coronaviruses. (B) Phylogenetic characterization of SARS-CoV-2 *ORF1AB* sequence (HEE1) inferred by maximum likelihood. The sequence was aligned to other SARS-CoV-2 strains and the closely related bat coronavirus strain RaTG13 downloaded from GenBank NCBI (www.ncbi.nlm.nih.gov).

Taxonomic classification of the sequences was performed using the Kaiju platform (4). Metagenome analysis yielded a total of 206,111 DNA sequences with 43,603,091 bases and a read length *N*_50_ value of 263 bp. Viral sequences represented 0.9% of the total metagenome, 4% of which corresponded to *Coronavirinae*. In this group, 83% represented nonassigned coronaviruses, and 17% were identified as SARS coronaviruses (Fig. 1A). Additionally, several bacterial and eukaryotic sequences related to the patient’s respiratory microbiota were identified. The most relevant taxa found were Streptococcus pneumoniae (7%), *Chlamydia* spp. (5%), Mycobacterium tuberculosis (4%), and Staphylococcus aureus (3%). We did not identify any particular clinically relevant fungus.

A 3,173-bp SARS-CoV-2 consensus sequence was obtained by mapping reads against the reference strain Wuhan-Hu-1 (GenBank accession number MN908947) using minimap2 version 2.14-r883 (5). Samtools version 1.9 (http://samtools.github.io) and Tablet alignment viewer version 1.19.09.3 (https://ics.hutton.ac.uk/tablet) were used to visualize the mapped sequence. A sequence similarity of 99.68% was found with *ORF1AB*, with 100% coverage. To confirm taxonomic classification, a phylogenetic tree (Fig. 1B) was inferred by using the maximum likelihood method and the Tamura-Nei model with MEGA X (6). The sequences used to build the phylogenetic tree included the *ORF1AB* gene sequence recovered from the metagenome analysis and the sequences of 7 SARS-CoV-2 strains and the closely related bat coronavirus strain RaTG13 (Fig. 1B) from GenBank NCBI (www.ncbi.nlm.nih.gov). All bioinformatic tools were run with default parameters.

Ethical approval for using the sample was given by CEISH-USFQ (Comité de Ética de Investigación en Seres Humanos-USFQ) (IE-JP067-2020-CEISH-USFQ).

### Data availability.

The metagenome sequences are publicly available at accession number PRJNA613094 (Fastq for called reads, SRR11341345; raw Fast5, SRR12664395).
